# The mother of all battles: Viruses vs humans. Can humans avoid extinction in 50–100 years?

**DOI:** 10.1515/biol-2022-0005

**Published:** 2022-01-29

**Authors:** Eleftherios P. Diamandis

**Affiliations:** Lunenfeld-Tanenbaum Research Institute, Mount Sinai Hospital, 60 Murray St [Box 32], Rm L6-201-1, Toronto, ON, M5T 3L9, Canada; Department of Laboratory Medicine and Pathobiology, University of Toronto, Toronto, Canada; Department of Pathology and Laboratory Medicine, Mount Sinai Hospital, Toronto, Canada; Department of Clinical Biochemistry, University Health Network, Toronto, Canada

**Keywords:** pandemics, contagious diseases, human race extinction, viruses, microbiome, COVID-19, blindness

## Abstract

The recent SARS-CoV-2 pandemic, which is causing COVID-19 disease, has taught us unexpected lessons about the dangers of human suffering through highly contagious and lethal diseases. As the COVID-19 pandemic is now being partially controlled by various isolation measures, therapeutics, and vaccines, it became clear that our current lifestyle and societal functions may not be sustainable in the long term. We now have to start thinking and planning on how to face the next dangerous pandemic, not just overcoming the one that is upon us now. Is there any evidence that even worse pandemics could strike us in the near future and threaten the existence of the human race? The answer is unequivocally yes. It is not necessary to get infected by viruses found in bats, pangolins, and other exotic animals that live in remote forests to be in danger. Creditable scientific evidence indicates that the human gut microbiota harbor billions of viruses that are capable of affecting the function of vital human organs such as the immune system, lung, brain, liver, kidney, or heart. It is remotely possible that the development of pathogenic variants in the gut can lead to contagious viruses, which can cause pandemics, leading to the destruction of vital organs, causing death or various debilitating diseases such as blindness, respiratory, liver, heart, and kidney failures. These diseases could result in the complete shutdown of our civilization and probably the gradual extinction of the human race. This essay will comment on a few independent pieces of scientific facts, and then combine this information to come up with some (but certainly not all) hypothetical scenarios that could cause human race misery, even extinction, in the hope that these hypothetical scenarios will trigger preventative measures that could reverse or delay the projected adverse outcomes.

## 1 Introduction

**Le Chatelier’s Principle:** Named after the French chemist, Le Chatelier’s principle posits that “*When an external stress (change in pressure, temperature or concentration) is applied to a system in chemical equilibrium, the equilibrium will change in such a way as to reduce the effect of the stress.*” In other words, a change in a system will evoke a counter-change, which will bring the equilibrium to a new point. This principle operates with almost every human or other activity. For example, it is known that when fruit production in the Serengeti ecosystem is reduced, the number of elephants, which feed on these fruits, is reduced proportionally. In the context of this essay, I hypothesize that human-made changes in climate, the atmosphere, water, soil, and all other planet-living organisms, will likely evoke counter-changes that may be highly consequential to human life. Due to the complexity of our ecosystem, humans do not know exactly how these changes will affect them in the end. Consequently, they choose to disregard them because lifestyle adjustments may cost money and convenience or loss of well-established pleasures.

### 1.1 The earth is changing rapidly

What is changing on the earth that could induce a potentially catastrophic counter-change? The answer is everything is changing 1Greek Philosopher Heraclitus (c. 535–475 bc) famously said πάντα ῥεῖ (panta rhei) “all is flux” or “everything flows.”, from the living inhabitants (humans, other species, and plants) to the atmosphere, water, soil, climate, among else.

The changes caused by human activity are sometimes dramatic. For example, it has been estimated that about 1 million out of 8.5 million species of plants, animals, and other organisms are in imminent danger of extinction [1]. Other estimates show that 50% of the organisms that existed 50 years ago have already gone extinct, not to consider additional species that are gone before we even identify them. Soon, we will likely be losing more than 80% of the world’s species due to human overdevelopment and its associated consequences. The major reasons for species extinction are habitat destruction, pesticide poisoning, and illegal hunting [1].

## 2 Global warming

Some may choose to believe what the politicians are debating about: that climate change is a fact or fiction, but the data say that the last 6 years were the warmest on record [2]. Overall, the planet was 1.25°C warmer than in preindustrial times (in the 1950s). Warmer oceans are melting ice sheets and rising sea levels by almost 5 mm per year. In Australia, record-setting heat and drought were responsible for the bushfires that destroyed almost 25% of southeastern Australia’s forests and their living inhabitants, such as koalas. If we cannot slow down earth’s heating by reducing emissions, the current increase of about 0.2°C per decade will likely be rapidly surpassed. How will the planet react? Likely with more catastrophic fires, tsunamis, earthquakes, and floods. The human homeostatic changes to increased temperatures are very complex and include many vital organs [3]. Global warming may also cause changes in the biology of our candidate foes, the viruses, bacteria, and parasites that live in our gut and skin (see Section 2.2).

### 2.1 How much human-made environmental damage has been done already?

Humans are now the undisputed masters of the planet and cannot be easily stopped from actively destroying it, consciously or unconsciously. An interesting question is how much damage has been claimed to be done already, and do we have the data to support these claims? Elhacham et al. have recently compared the natural biomass that exists on the earth with the human-made (anthropogenic) mass [4]. They found that each person on the globe produces a mass that is about equal to their body weight every week! Is that too little or too much? Let us first define biomass and anthropogenic mass. The majority of the earth’s biomass is represented by trees and bushes. The majority of the man-made mass is represented by buildings and infrastructure such as roads and consists of concrete, bricks, asphalt, metals, and plastic. Just consider that the total global mass of produced plastic so far is greater than the overall mass of all terrestrial and marine animals combined!

So, how do we fare when comparing biomass to anthropogenic mass production? In the 1900s, the latter represented only 3% of global biomass; but now, in the 2020s, the two masses are about equal. The projection is that if we go on with more deforestation, buildings, streets, plastics, cars, and so on, by 2040, it is likely that anthropogenic mass will almost triple the earth’s biomass. Will there be enough resources and clean air and water to sustain the life of the projected 9 billion inhabitants? Anthropogenic mass production is difficult to slow down since this activity is considered part of our evolving civilization and way of living.

### 2.2 Human microbiome

The human body consists of approximately 30 trillion cells, but the microbiota population in the human gut is estimated to be 300 trillion [5]! In addition, there is another microbiota in the skin and other organs. It was initially thought that these microbiota act locally (e.g., only in the gut or skin), but new evidence suggest that the effects of microbiota may be global, reaching every cell in the body. This can be achieved with various mechanisms, one being the transmission of signals mediated by proteins that can travel through anatomically distinct structures such as the vagus nerve. For example, a protein called *curli* can travel through the vagus nerve and reach the brain, where it can promote abnormal aggregation of proteins such as a-synuclein, one major pathogenetic player in Parkinson’s disease [5,6]. Another and even more likely mechanism includes the diffusion of bacterial or viral proteins (some could be toxins to various organs) or pathogenic viruses into the bloodstream. From there, they can travel around the body. This is reminiscent of cancer cell metastasis by the hematogenous route. One piece of evidence for that happening is that about half of the human metabolome (the collection of all metabolites in the blood) is derived by host bacteria [5]. Bacteria or virus-derived metabolites could also pass through the placenta and reach the fetus, including the fetal brain, possibly causing diseases such as autism.

Despite skin not being as hospitable to microorganisms as the gut, a typical person may have about 1,000 species of bacteria on their skin [7]. These microbial communities continue to grow and diversify until puberty when hormonal and developmental changes reach a plateau. The balance between host and bacteria in the skin is determined by the production of skin-derived microbial nutrients, microbiome-derived skin nutrients, skin, and microbiome-derived antimicrobial peptides, and by the interaction of the microbiome with the host’s immune system. Similar as in the gut, there is a delicate balance between beneficial and potentially harmful bacteria and the host immune system. It is remotely possible that our future enemies may derive from the gut, skin, or other organs harboring microorganisms. In addition, the skin is more sensitive to environmental changes such as climate change as it is directly exposed to the environment.

In conclusion, bacterial, viral, and parasite-derived proteins or pathogenic viruses thrive locally (e.g., in the gut or skin) but are capable of acting globally.

### 2.3 Human viruses and how they could cause disease

Many strains of gut bacteria are harmless, but they can become dangerous pathogens under certain conditions, such as antibiotic use [8]. It is well known that gut bacteria can harbor many viruses (bacterial phages) [9]. If they do not immediately kill the infected bacteria, these viruses incorporate into the bacterial genome and stay latent for extended periods (they are known “prophages”). These prophages can be reactivated under certain environmental or other factors and act like pathogenic viruses. It is rather surprising that, in general, viruses are so many that they qualify as the most abundant biological entities on the planet. Sometimes, gut bacteria use their activated prophages as weapons to gain an advantage and kill other competing bacteria. Phages could also assist in bacterial evolution as the latter become more virulent [10]. The gut bacteria also seem to interact with the host immune system and can influence the efficacy of cancer immunotherapy [11,12,13]. The microbiome has been blamed for playing direct or indirect roles in many human diseases, including cancer, metabolic syndrome, diabetes, dementia, and others [14].

The outcomes regarding health and disease depend on the balance of powers among the gut/skin/other organ viruses, the gut/skin/other organ microbiomes, and the host immune system. If this balance is disturbed, a biological war between these players will be initiated, and the outcome will be unpredictable.

In conclusion, scientific evidence supports the idea that phages in the mammalian intestine, skin, or elsewhere, not only can be engulfed by certain eukaryotic cells but also might escape from the gut or skin, enter the bloodstream, and make their way into other parts of the body, with as yet undiscovered consequences.

### 2.4 Viral variants

Viruses evolve continuously, eventually leading to more transmissible variants, which sometimes can be more lethal than the original strains. The SARS-CoV-2 is an excellent contemporary example. Multiple variants of SARS-CoV-2 are rapidly spreading and are becoming dominant in certain geographic areas [15,16]. For example, the B.1.1.7 variant (United Kingdom) has 23 mutations and 17 amino acid changes; variant 501Y.V2 (South Africa) has 23 mutations and 17 amino acid changes; and P.1 variant (Brazil) has approximately 35 mutations with 17 amino acid changes.

In April 2021, when this document was first written, I speculated verbatim that “new variants with additional mutations could become able to evade our currently available vaccines by weakening the ability of vaccine-induced antibodies to neutralize/block viral entry, and by strengthening the ability of the virus to enter the cells via surface receptors.” The so-called “omicron variant,” isolated in November 2021, already fulfilled this prediction.

### 2.5 How COVID-19 and possibly other viruses affect the brain

In general, viral invasion of the central nervous system may be achieved by several routes, including transsynaptic transfer across infected neurons, entry via the olfactory nerve, infection of vascular endothelium, or leukocyte migration across the blood–brain barrier. SARS-CoV-2 invades endothelial cells via transmembrane angiotensin-converting enzyme 2 (ACE2) receptor binding and a subsequent proteolytic event, facilitated by transmembrane protease serine 2 [17]. Is there evidence that SARS-CoV-2 can enter the brain? The answer is yes [18]. As already mentioned, one route is by migrating from the cribriform plate along the olfactory tract [19] or through vagal pathways. Another route may include viral entry into brain capillary endothelial cells via the ACE2 pathway. Viral RNA was detected in the medulla and cerebellum by reverse transcription-polymerase chain reaction. However, viral proteins seem to be absent from neurons and glial cells. Consequently, the adverse events of the virus on the brain, including altered neurotransmission and neuronal damage, are likely mediated by neuroinflammation and hypoxic injury through cytokines and other proinflammatory mediators.

### 2.6 SARS-CoV-2 and possibly other viruses can affect the senses

Viruses can affect our senses. For example, SARS-CoV-2 causes anosmia (loss of smell) and ageusia (loss of taste) in 40–70% of COVID-19 patients [20]. These effects persist, but it is unknown for how long. Other neurological symptoms include headache, stroke, impairment of consciousness, seizure, anxiety, and encephalopathy.

Current evidence suggests that SARS-CoV-2-related anosmia may be a new viral syndrome specific to COVID-19. This syndrome is likely mediated by intranasal inoculation of SARS-CoV-2 into the olfactory neural circuitry. Since the olfactory sensory neurons do not express ACE2 receptor, the likely explanation for the loss of smell is the damage of accessory cells supporting these neurons.

Although anosmia is not a lethal or severe disease, other neurological damage such as blindness could be devastating [21,22].

## 3 Adverse scenarios

Fifty years ago, one adverse scenario regarding a pandemic was presented in the film “The Andromeda strain,” which describes a pandemic caused by a pathogen of extraterrestrial origin [23]. Here, I present an alternative hypothetical scenario that involves an endogenous virus. Obviously, there is a myriad of similar scenarios, and the one given below can be currently classified as fictional but not impossible.

A prophage, which was residing dormant for years in the genome of the commensal gut bacterium *Bifidobacterium infantis* suddenly, and without an apparent reason, has undergone induction and started to produce viral proteins, which were subsequently assembled into whole phages. After cell lysis, these phages infected other neighboring cells. This cycle was repeated many times, and millions of free virions were released, some entering the systemic circulation (viremia). Some virions reached the lung endothelium and entered the endothelial cells through an, as yet, unknown receptor and started replicating and lysing these cells. The resulting mucous caused the host to cough, thus facilitating the transfer of the virus to other humans through aerosol droplets. Soon, the virus was able to infect, first a few hundred, then thousands, then millions of other unsuspected people through coughing and sneezing. The virus was able to travel all over the world as the pulmonary manifestations were mild, and most infected individuals thought it was a common flu or a similar ailment.

Scientists isolated the virus that caused this flu-like disease and determined from its genomic sequence that it was a novel member of influenza virus B, which usually causes seasonal flu. Despite the pandemic nature of the infection, nobody died, and governmental bodies were not highly concerned.

Six months later, one individual reported a weakening of his vision, which, within 3 months, progressed to total blindness. This unusual form of blindness quickly spread to other people until scientists performed epidemiological studies, which linked the blindness to the previously mentioned mild flu. Soon afterward, scientists isolated and identified the virus from the brains of blind and subsequently succumbed individuals and confirmed that the sequence matched the virus that caused the unusual flu. More elaborate studies had shown that there was unusual and very severe neuroinflammation around the occipital lobe of the brain (Brodmann area 17), an area responsible for the interpretation of visual signals arriving from the optic nerve. Several therapeutics were tried, but none was proven to be effective. Twelve months into the pandemic, 10 million people lost their vision, and within 18 months, without any success in developing therapies or a vaccine, the blindness had spread to whole nations.

### 3.1 Blindness

The selection of blindness as a chronic consequence of an acute pandemic was deliberate. In 1995, Portuguese author Jose Saramago published a fictional novel entitled “Blindness” (ISBN: 9780151002511), which contributed to him winning the Nobel Prize in literature in 1998.

Blindness, as portrayed in the book, is a highly detailed story of a mysterious mass epidemic that caused blindness of a whole nation and the social breakdown that followed. The blindness pandemic, in many respects, is reminiscent of the current COVID-19 pandemic. Blindness caused widespread panic, anarchy, and government lockdowns. The life of the blind people was characterized by filthiness, aggressive manners, disrespect of others, and a struggle to survive by any possible means. The breakdown of society was near total. Law and order, social services, government, schools could no longer function. Families have been separated and could not find one another. People squat in abandoned buildings and scrounge for food. Violence, disease, and despair threaten to overwhelm human coping. One of Saramago’s quotes, describing life after blindness, is reproduced here

“Perhaps humanity will manage to live without eyes, but then it will cease to be humanity, the result is obvious…”

### 3.2 Other ailments

Acute pandemics could cause many other chronic diseases that can threaten the sustainability of our present society. Although COVID-19 causes loss of smell and taste, these are considered nonlife-threatening ailments. However, in the long run, the permanent absence of smell and taste will mean the loss of innumerable current pleasures associated with the consumption of food and drinks. Clearly, loss of hearing will not be compatible with current societal functions or human achievements. Acute viral diseases are also associated with innumerable organ-specific diseases such as heart, kidney, and reproductive failures and disturbance of other vital functions that can paralyze our current society, economy, and culture. Even a minor weakening of our memory (mild cognitive impairment) could result in chaotic situations that authors of fiction, such as Saramago, attempt to describe in detail in future books.

### 3.3 Epilog

Humans have learned to take for granted what they currently have and enjoy. Perhaps, we did not realize that the human race’s spectacular advances are dependent on several potentially volatile abilities (senses, brain function) and that even one loss, or diminution of such abilities, could be detrimental, causing a collapse of our civilization. The COVID-19 pandemic helped us realize that we may be sitting on a time bomb, which might explode if we continue disturbing the current equilibrium between humans and other planetary partners. In addition to viruses of a rather exotic origin, such as SARS-CoV-2, billions of other viruses and other infectious agents in our gut, skin, and elsewhere are waiting for the right time to attack us. The lessons learned from COVID-19 should be a wake-up call for humans to stop disturbing the equilibrium with actions that favor the well-being of humans but put in danger the existence of other inhabitants of planet earth. Human migration, also known as “travel,” has facilitated the travel of our foes, along with us, in every conceivable corner of the world.

Finally, yet, importantly, artists are always ahead of scientists in seeing things coming. On this occasion, the rock band R.E.M. released a song 30 years ago entitled “It’s the end of the world as we know it (and I feel fine).” They are likely not far off their prediction!
